# Metabolic Syndrome in Older Adults: When Function Trumps Numbers

**DOI:** 10.17925/EE.2025.21.2.6

**Published:** 2025-09-22

**Authors:** Marcio José Concepción-Zavaleta, Eduardo Cabellos-Acuña, Jenyfer Maria Fuentes-Mendoza, José Paz-Ibarra

**Affiliations:** 1. School of Medicine, Universidad Científica del Sur, Lima, Peru; 2. Department of Geriatrics, Hospital de Emergencias Villa El Salvador, Lima, Peru; 3. Department of Endocrinology, Hospital Nacional Edgardo Rebagliati Martins, Lima, Peru; 4. Facultad de Medicina de la Universidad Nacional Mayor de San Marcos, Lima, Peru

**Keywords:** Ageing, frailty, hyperglycaemia, hypertension, metabolic syndrome, obesity, sarcopenia

## Abstract

This editorial critiques the limitations of traditional metabolic syndrome criteria in older adults, arguing that age-related changes in body composition, vascular stiffness and frailty render standard thresholds inadequate. Highlighting the ‘obesity paradox’ and risks of intensive blood pressure/lipid control, the article advocates for a frailty-centred approach integrating functional status, body composition (e.g. dual-energy X-ray absorptiometry) and inflammation markers over rigid numerical targets. Practical strategies include sarcopenia screening (SARC-F), resistance training and individualized glycaemic/blood pressure goals to avoid overtreatment in frail patients. The paradigm shift aims to improve risk stratification and align care with geriatric priorities.

The metabolic syndrome (MetS) construct, while valuable for middle-aged populations, shows critical limitations when applied to older adults. Traditional criteria emphasizing waist circumference, blood pressure, lipids and glucose fail to capture the complex interplay between ageing physiology and metabolic dysfunction.^1,2^ This oversight becomes clinically significant when considering that up to 30–40% of older adults with normal body mass index (BMI) have sarcopenic obesity, a condition where muscle loss coexists with excess adiposity.^3,4^ The consequences are profound: sarcopenic obesity confers a 2.6-fold higher risk of physical disability compared with obesity alone; yet, it remains invisible to standard MetS diagnostics.^5^ These gaps demand a paradigm shift towards frailty-centred assessment, where functional status and body composition take precedence over rigid numerical thresholds (*Figure 1*).

The limitations of current MetS criteria become especially apparent when examining anthropometric measures. While BMI thresholds identify obesity in younger populations, ageing alters body composition in ways that render BMI misleading for older adults.^3^ This limitation is particularly significant in Asian populations, where lower BMI thresholds (≥27.5 kg/m² versus ≥30 kg/m² in Caucasians) indicate equivalent metabolic risk due to ethnic variations in body composition.^5,6^

Sarcopenic obesity (the combination of low muscle mass and high adiposity) has been shown to be a stronger predictor of mortality than BMI alone in the elderly.^3^ For clinical assessment, dual-energy X-ray absorptiometry scanning provides comprehensive body composition analysis, while in resource-limited settings, practical alternatives include calf circumference (<31 cm) or handgrip strength (<27 kg for men/<16 kg for women) as validated screening tools for sarcopenia risk.^4^

Standard anthropometric markers fail to capture sarcopenic obesity, a key driver of disability in ageing populations. Epidemiological studies highlight this disconnect through the ‘obesity paradox’, where older adults with BMI around 28 kg/m² (the point of lowest mortality risk in Mendelian randomization studies) sometimes outperform normal-weight peers in survival outcomes.^7^ This paradox stems from BMI’s inability to differentiate muscle mass from fat mass, particularly seen as a problem in ethnic groups with distinct body composition phenotypes. Together, these findings demand ethnicity- and age-adjusted approaches to metabolic risk assessment.

Similar challenges apply to other MetS components. Hypertension management based on universal targets (<130/80 mmHg) derived from middle-aged trials may be inappropriate for older adults with vascular stiffening and impaired cerebral autoregulation.^8^ While trials like the Systolic Blood Pressure Intervention Trial Senior (SPRINT-SENIOR; ClinicalTrials.gov identifier: NCT01206062) showed increased adverse events (including falls and renal complications) in vulnerable subgroups, as shown in subgroup analyses, the Hypertension in the Very Elderly Trial (HYVET; ClinicalTrials.gov identifier: NCT00122811), targeting <150 mmHg, demonstrated both safety and cardiovascular benefits of moderate control.^8,9^ This clinical dichotomy supports individualized targets (e.g. <140–150 mmHg systolic in frail patients) that balance vascular protection with functional safety.

**Figure 1:**
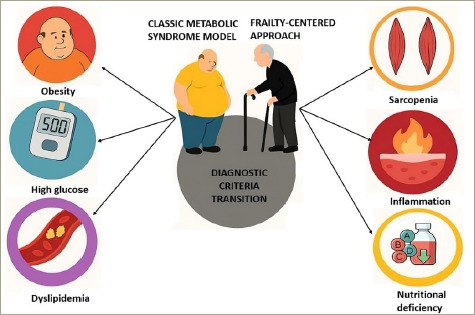
Transition from the classical metabolic syndrome model to a frailty-centred approach in older adults

The ‘lipid paradox’, where low cholesterol correlates with higher mortality in the oldest adults, suggests that lipid levels may reflect frailty and inflammation rather than cardiovascular risk in this population.^10^ These nuances underscore why metabolic parameters must be interpreted through the lens of biological age and functional reserve.

Frailty provides the missing link in this assessment. Defined as decreased physiological reserve and resistance to stressors, frailty captures the intersection between metabolic dysfunction and functional decline.^11^ The Fried frailty phenotype, incorporating unintentional weight loss, exhaustion, slow gait, low physical activity and weak grip strength, identifies vulnerability that the traditional MetS criteria miss.^11^ Critically, detecting pre-frailty (e.g. 1–2 Fried criteria) enables early interventions like protein supplementation and resistance exercise, which may prevent progression to full frailty.^12^ Importantly, frailty and MetS share underlying mechanisms, particularly chronic low-grade inflammation (‘inflamm-ageing’) and insulin resistance.^13^ Elevated inflammatory markers like interleukin (IL)-6 and C-reactive protein (CRP) not only drive muscle catabolism but also exacerbate metabolic dysfunction, creating a vicious cycle that accelerates both frailty and cardiometabolic decline.^14^ This biological synergy explains why older adults with both MetS and frailty face 2.7-fold higher mortality than those with either condition alone.^15^

Practical implications of this frailty-metabolism interplay are most evident in therapeutic decisions. Where middle-aged MetS management emphasizes aggressive glycaemic control and statin therapy, geriatric care requires balancing metabolic targets against functional preservation.^16^ While tight control should be avoided, persistent hyperglycaemia (>200 mg/dL fasting) accelerates cognitive decline and disability; individualized goals (glycated haemoglobin 7–8.5%) based on frailty status are recommended.^17^ Resistance exercise combined with protein supplementation, for example, may improve muscle mass and insulin sensitivity more effectively than medication intensification in sarcopenic older adults.^16^

Similarly, deprescribing antihypertensives or statins may be appropriate in frail patients where polypharmacy risks outweigh potential benefits.^18^ The Screening Tool of Older Persons’ Prescriptions/Screening Tool to Alert to Right Treatment (STOPP/START) criteria provide a framework for such medication optimization, though clinical judgement remains essential.^19^ Emerging tools like the Cardiometabolic Index (CMI), which integrates waist-to-height ratio with lipid profiles, may eventually bridge metabolic and functional assessments but require validation in diverse geriatric populations.^20^

Moving forward requires the development of validated, integrated screening tools that combine functional assessments (gait speed or SARC-F), accessible body composition measures (calf circumference or bioimpedance) and metabolic-inflammation markers (waist-to-height ratio or CRP), with adjustments for ethnic-specific thresholds in diverse geriatric populations.^13,17^ These tools should undergo rigorous testing across clinical settings, from specialized geriatric units to primary care in resource-limited regions, while maintaining predictive validity comparable to gold-standard methods. The Clinical Frailty Scale offers a simple way to incorporate frailty assessment into routine metabolic evaluations.^21^

In conclusion, the traditional MetS framework serves younger populations well but falters in geriatric care. By integrating frailty assessment with metabolic evaluation, clinicians can better identify high-risk older adults and tailor interventions that prioritize function and quality of life. This evolution aligns with geriatrics’ core principle: adding life to years, not just years to life.

## References

[1] Reaven GM (1993). Role of insulin resistance in human disease (syndrome x): An expanded definition.. Annu Rev Med..

[2] Grundy SM, Cleeman JI, Daniels SR (2006). Diagnosis and management of the metabolic syndrome: An American Heart Association/National Heart, Lung, and Blood Institute scientific statement.. Curr Opin Cardiol..

[3] Batsis JA, Mackenzie TA, Barre LK (2014). Sarcopenia, sarcopenic obesity and mortality in older adults: Results from the National Health and Nutrition Examination Survey III.. Eur J Clin Nutr..

[4] Cruz-Jentoft AJ, Bahat G, Bauer J (2019). Sarcopenia: Revised European consensus on definition and diagnosis.. Age Ageing..

[5] Baumgartner RN, Wayne SJ, Waters DL (2004). Sarcopenic obesity predicts instrumental activities of daily living disability in the elderly.. Obes Res..

[6] WHO Expert Consultation. (2004). Appropriate body-mass index for Asian populations and its implications for policy and intervention strategies.. Lancet..

[7] Lv Y, Zhang Y, Li X (2024). Body mass index, waist circumference, and mortality in subjects older than 80 years: A Mendelian randomization study.. Eur Heart J..

[8] Lee D-H, Lee J-H, Kim SY (2022). Optimal blood pressure target in the elderly: Rationale and design of the HOW to Optimize eLDerly systolic Blood Pressure (HOWOLD-BP) trial.. Korean J Intern Med..

[9] Beckett NS, Peters R, Fletcher AE (2008). Treatment of hypertension in patients 80 years of age or older.. N Engl J Med..

[10] Ravnskov U, Diamond DM, Hama R (2016). Lack of an association or an inverse association between low-densitylipoprotein cholesterol and mortality in the elderly: A systematic review.. BMJ Open..

[11] Fried LP, Tangen CM, Walston J (2001). Frailty in older adults: Evidence for a phenotype.. J Gerontol A Biol Sci Med Sci..

[12] Apóstolo J, Cooke R, Bobrowicz-Campos E (2018). Effectiveness of interventions to prevent pre-frailty and frailty progression in older adults: A systematic review.. JBI Database Syst Rev Implement Rep..

[13] Fulop T, Larbi A, Dupuis G (2017). Immunosenescence and inflamm-aging as two sides of the same coin: Friends or foes?. Front Immunol..

[14] Beavers DP, Kritchevsky SB, Gill TM (2021). Elevated IL-6 and CRP levels are associated with incident self-reported major mobility disability: A pooled analysis of older adults with slow gait speed.. J Gerontol A Biol Sci Med Sci..

[15] Kojima G (2018). Frailty defined by FRAIL scale as a predictor of mortality: A systematic review and meta-analysis.. J Am Med Dir Assoc..

[16] Liao C-D, Tsauo J-Y, Wu Y-T (2017). Effects of protein supplementation combined with resistance exercise on body composition and physical function in older adults: A systematic review and meta-analysis.. Am J Clin Nutr..

[17] Strain WD, Down S, Brown P (2021). Diabetes and frailty: An expert consensus statement on the management of older adults with type 2 diabetes.. Diabetes Ther..

[18] Ekram ARMS, Woods RL, Ryan J (2022). The association between polypharmacy, frailty and disability-free survival in community-dwelling healthy older individuals.. Arch Gerontol Geriatr..

[19] Petrazzuoli F, Morin L, Angioni D, Demurtas J, Veronese N (2022). The Role of Family Physicians in Older People Care. Practical Issues in Geriatrics..

[20] Luo Y, Yin Z, Li X (2025). Cardiometabolic index predicts cardiovascular events in aging population: A machine learning-based risk prediction framework from a large-scale longitudinal study.. Front Endocrinol..

[21] Rockwood K, Song X, MacKnight C (2005). A global clinical measure of fitness and frailty in elderly people.. CMAJ..

